# Repositioning of the anthelmintic drug mebendazole for the treatment for colon cancer

**DOI:** 10.1007/s00432-013-1539-5

**Published:** 2013-10-18

**Authors:** Peter Nygren, Mårten Fryknäs, Bengt Ågerup, Rolf Larsson

**Affiliations:** 1 Division of Clinical Pharmacology, Department of Medical Sciences, Uppsala University, 751 85 Uppsala, Sweden; 2 Section of Oncology, Department of Radiology, Oncology and Radiation Science, Uppsala University, 751 85 Uppsala, Sweden; 3 NX2B, Uppsala, Sweden

**Keywords:** Repositioning, Colon cancer, Therapy, Benzimidazoles, Mebendazole

## Abstract

**Purpose:**

In the present study, we screened a compound library containing 1,600 clinically used compounds with the aim to identify compounds, which potentially could be repositioned for colon cancer therapy.

**Methods:**

Two established colon cancer cell lines were tested using the fluorometric microculture cytotoxicity assay (FMCA). For compound comparison connectivity map (CMAP) analysis, NCI 60 data mining and protein kinase binding measurements were performed.

**Results:**

Sixty-eight compounds were defined as hits with activity in both of these cell lines (<40 % cell survival compared with control) at 10 μM drug concentration. Analysis of chemical similarity of the hit compounds revealed several distinct clusters, among them the antiparasitic benzimidazole group. Two of these compounds, mebendazole (MBZ) and albendazole (ABZ) are registered for human use. Data from the NCI 60 cell line panel revealed only modest correlation between MBZ and ABZ, indicating differences in mechanism of action. This was further supported when gene expression signatures were compared in the CMAP database; ABZ ranked very low when MBZ was used as the query signature. Furthermore, MBZ, but not ABZ, was found to significantly interact with several protein kinases including BCR–ABL and BRAF. Analysis of the diagnosis-specific activity of MBZ showed activity in 80 % of the colon cancer cell lines in the NCI 60 panel. Three additional colon cancer cell lines and three cell models with non-malignant phenotypes were subsequently tested, confirming selective colon cancer activity of MBZ.

**Conclusion:**

MBZ seemingly has repositioning potential for colorectal cancer therapy.

## Introduction

In the past decades, target-based approaches with the aim to modulate specific diagnosis-related molecules and pathways have dominated the cancer drug discovery process and resulted in several successful novel therapies, best exemplified by the bcr–abl inhibitor imatinib for the treatment for chronic myelocytic leukemia (CML) (Swinney and Anthony 2011). However, this approach is slow, has a high failure rate and it has become obvious that the rate of new drug approvals is decreasing (Hurle et al. 2013; Sukhai et al. 2011). Furthermore, the compounds that reach the market produce modest benefit in solid tumors (Hurle et al. 2013). Thus, new strategies for drug discovery are needed. One such strategy is drug repositioning (or repurposing) in which a new indication for an existing drug is identified. In this approach, known on-patent, off-patent, discontinued and withdrawn drugs with unrecognized cancer activity can be rapidly advanced into clinical trials for this new indication as much or all of the required documentation to support clinical trials can rely on previously published and readily available data (Hurle et al. 2013).

Genomics-based target identification and screening using cell-free systems have been the dominating principle in cancer drug discovery during the recent decade (Mayr and Bojanic 2009). As an alternative to this approach, the use of phenotype-cell-based screening using human tumor cell lines may provide some distinct advantages (van Staveren et al. 2009). Cellular phenotype results from many interconnecting signaling and feedback pathways. Compound screens based on isolated targets or cell components do not reflect this complexity. Indeed, it has recently been shown that the contribution of phenotypic screening to the discovery of first-in-class small molecule drugs exceeded that of target-based approaches (Swinney and Anthony 2011). Furthermore, recent findings from large panels of cell lines indicate that they retain genomic features of the primary tumor and can recapitulate clinical findings with regard to response to targeted inhibitors (Sharma et al. 2010).

In the present study, we used an annotated library containing 1,600 compounds with documented clinical use for screening in colon cancer cell lines with the aim to identify drugs with repositioning potential in this cancer type.

## Methods

### Cell culture

For primary screening, the colon cancer cell lines HCT 116 and RKO, obtained from American Type Culture Collection (ATCC; Rockville, MD, USA), were cultured in McCoy’s 5a medium supplemented with 10 % heat-inactivated fetal bovine serum, 2 mM l-glutamine, 100 μg/ml streptomycin and 100 U/ml penicillin (all from Sigma-Aldrich St. Louis, MO, USA).

The colon cancer cell lines HT29, HT-8 and SW626 and the normal phenotype non-malignant epithelial MCF 10A, renal RPTEC/TERT1 and hepatocyte NeHepLxHT cell lines were used for validation experiments. These cell lines were all obtained from ATCC and cultured according to the protocols provided by the supplier. All the cell lines were cultured at 37 °C in a humidified atmosphere containing 5 % CO_2_.

### Preparation of compounds for screening

The Pharmakon 1600 library (Microsource Inc, MO, USA) consists of 1,600 annotated compounds previously used in the clinical setting. The library was delivered in 36 racks each containing 80 compounds at a concentration of 10 mM dissolved in DMSO. For the primary screening, aliquots of the DMSO solutions were transferred to 384-well Labcyte source plates (Labcyte Inc, CA, USA) using a Biomek 2000 and stored at room temperature in containers under nitrogen atmosphere. These source plates were subsequently used to deliver 10 μM (final concentration) of each drug to 384-well screening plates using a acoustic dispensing Echo 550 instrument equipped with Labcytes Access robotics (Labcyte).

For dose–response studies, Labcyte source plates and the Echo dispenser were used in a similar manner to deliver different concentrations of each drug to destination plates using on board software protocols (Labcyte).

### Measurement of cancer drug activity

The fluorometric microculture cytotoxicity assay (FMCA), described in detail previously (Lindhagen et al. 2008), was used for measurement of the cytotoxic effect of library compounds and the established standard drugs. The FMCA is based on measurement of fluorescence generated from hydrolysis of fluorescein diacetate (FDA) to fluorescein by cells with intact plasma membranes. Cells were seeded in 384-well plates using the pipetting robot Precision 2000 (Bio-Tek Instruments Inc, Winooski, VT) and cultured over night before drugs were added by the Echo 550 system. The number of cells per well was 2,500–5,000 adjusted individually for each cell line. In each plate, two columns without drugs served as controls and one column with medium only served as blank. Results are expressed as survival index (SI), defined as fluorescence of experimental wells in per cent of unexposed control wells with blank values subtracted.

### Gene expression analysis in the connectivity map

The drug-induced gene expression perturbations of MBZ and ABZ were studied using the connectivity map (CMAP) build 02 (www.broad.mit.edu/cmap) that contains genome-wide expression data for 1,309 compounds (Lamb et al. 2006). Since both ABZ and MBZ are present in the database, the similarity in gene expression can be compared. The query was based on the genes (probes) with >twofold up- (31 probes) or down (70 probes)-regulation after treatment with MBZ in MCF-7 cells (used since the most complete data set is on MCF-7). CMAP score is a measure of correlation (−1 and +1) using Kolmogorov-Smirnov statistics between the query signature and all other gene expression signatures in the CMAP database (Lamb et al. 2006).

### Kinase assay

The binding affinities of ABZ and MBZ were tested at 10 μM in a high-throughput binding assay (KINOMEscan, Discoverx, CA, USA) against a panel of 97 kinases tagged with DNA (Davis et al. 2011). Compounds that bind the kinase active site and directly or indirectly prevent kinase binding to an immobilized ligand will reduce the amount of kinase captured on solid support. Conversely, test molecules that do not bind the kinase have no effect on the amount of kinase captured on the solid support. Hits are identified by measuring the amount of kinase captured in test versus control samples by using a quantitative PCR method that detects the associated DNA label (http://www.discoverx.com/tools-resources/publications-references). Results are presented as percent of control (POC). Selectivity score (S-score) is a quantitative measure of compound selectivity. S(35) is calculated as number of non-mutant kinases with POC <35/number of non-mutant kinases tested. Binding constants were determined with the same method using 11-point threefold serial dilutions of the compound. Curves were fitted using a nonlinear least square fit with the Levenberg–Marquardt algorithm (Davis et al. 2011).

### Data analysis and statistics

Screening data were exported to Vortex (Dotmatics Inc, UK) software for analysis. A SI of <40 % in both colon cancer cell lines was set as the criteria for qualifying as a hit compound. Structural similarity to other compounds in the library was calculated based on a structural fingerprint consisting of chemical fragments located within the compound as computed by Vortex. Clustering of compounds based on these fingerprints was then performed within the Vortex program.

Concentration–response data of screening hits and standard agents were analyzed using the software GraphPad Prism4 (GraphPad Software Inc., San Diego, CA, USA). Data were processed using nonlinear regression to a standard sigmoidal dose–response model to obtain IC_50_-values (the drug concentration resulting in a SI of 50 %).

Analysis of data from the National Cancer Institute growth inhibitory screen in 60 cell lines (NCI 60 GI 50 data) was performed with the help by the NCI Cellminer database (http://discover.nci.nih.gov/cellminer/). *Z* scores are determined for each experiment/cell line pair by the subtraction from its intensity by the experiment mean (across the 60 cell lines) and division by the standard deviation of the experiment (across the 60 cell lines). The z score average was then calculated as the mean across all experiments that passed quality control criteria. The *z* score will have a mean of zero and a standard deviation of one.

## Results and discussion

**Fig. 1:**
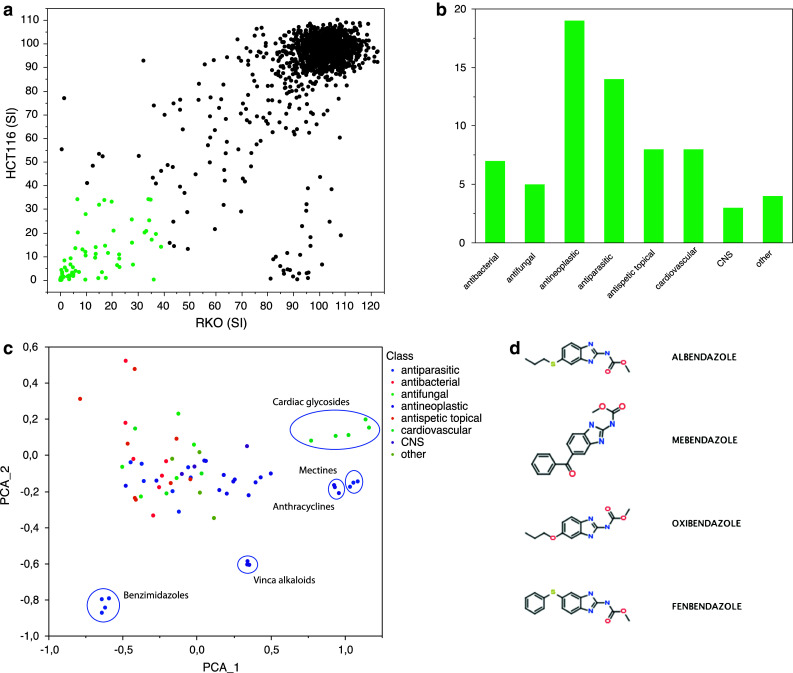
Screening for anticancer drug activity. The overall screening results are displayed and expressed as survival index. The *symbols* in *green* indicate the hit compounds (**a**). In **b,** the frequency of the major different pharmacological classes for the hit compounds is shown. Clustering of compounds based on chemical structures using Vortex principal component analysis (PCA) is shown in **c,** and some of the clusters are highlighted including the benzimidazole group of compounds. The chemical structures of the hit benzimidazole compounds are shown in **d**

**Fig. 2:**
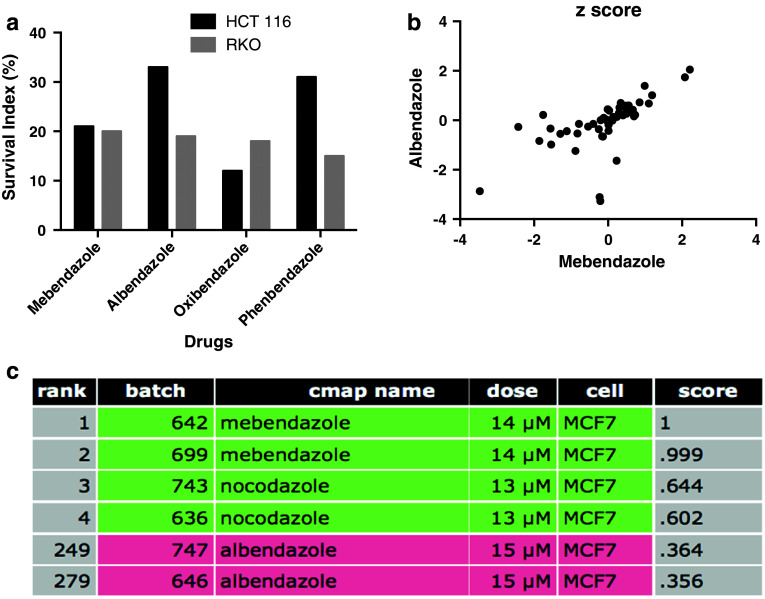
In **a,** the activity of the benzimidazole hit compounds using the FMCA is shown. The results are presented as survival index. The correlation between MBZ and ABZ *z* scores in the NCI 60 cell line panel is shown in **b**. Pearson’s correlation coefficient was 0.64. In **c,** CMAP results based on MBZ exposed MCF7 cells are shown. The CMAP score is a measure of similarity based on gene expression

The cytotoxic/antiproliferative activities in response to the 1,600 annotated compounds with documented clinical history at a concentration of 10 μM in the colon cancer cell lines RKO and HCT116 are shown in Fig. 1a. Sixty-eight hits, as defined above, were retrieved (4.25 % hit rate). Among the hits retrieved, the highest frequency was obtained for known antineoplastic agents followed by anti-parasitic drugs, anti-infectives and cardiovascular drugs (Fig. 1b). Clustering the compounds according to chemical structure using Vortex revealed clusters with similar chemical structure including anthracyclines, vinca alkaloids and cardiac glycosides (Fig. 1c). Interestingly, we also observed a distinct cluster containing the antihelmintic benzimidazole compounds albendazole (ABZ), mebendazole (MBZ), oxibendazole and fenbendazole (Fig. 1d). This group of compounds were within 2 standard deviations of the library with respect to log P, molecular weight, hydrogen donors and hydrogen acceptor, and were thus well in agreement with the Lipinski’s rule of 5 criteria (not shown). Oxibendazole and MBZ showed the lowest SIs in the two cell lines screened closely followed by ABZ and fenbendazole (Fig. 2a). From a repositioning perspective, MBZ and ABZ are registered pharmaceuticals for clinical use in humans, thus easily accessible for clinical testing, and were therefore prioritized for further analysis.

ABZ has previously demonstrated activity in several cancer types including colon cancer (Králová et al. 2013; Pourgholami et al. 2001, 2005). MBZ on the other hand has not previously been reported to be active in this disease and may have advantages both with respect to toxicity and efficacy. Thus, ABZ appears to have a more severe toxicity profile, including neutropenia and anemia (http://bioinformatics.charite.de/promiscuous/, von Eichborn et al. 2011). In a clinical study of patients with advanced cancers, ABZ-induced neutropenia (Morris et al. 2001) and neutropenia was also the dose-limiting toxicity in a subsequent formal dose-escalating phase-I trial (Pourgholami et al. 2010). ABZ has been reported to be redox active leading to strong reactive oxygen species (ROS) generation (Locatelli et al. 2004) potentially adding to the reported drug-induced toxicity. ROS generation could lead to several toxicological consequences as a result of oxidative injury to important biomolecules such as DNA, proteins and lipids (Kalinowski and Richardson 2007). In contrast, MBZ, although belonging to the benzimidazole group of compounds, showed only a low and transient effect on ROS generation (Locatelli et al. 2004). This suggests that MBZ could be the first-choice drug since it causes only a mild oxidative stress and thus is expected to better tolerate in the clinical setting.

Also from the standpoint of efficacy, MBZ may have advantages as demonstrated in a study of preclinical glioma models (Bai et al. 2011). In this study, MBZ, but not ABZ, was able to significantly increase survival in two orthotopic mouse models of glioma (Bai et al. 2011). In this context, it is worth noting that ABZ and MBZ activity patterns show only moderate correlations in the NCI60 panel (Pearson’s correlation coefficient 0.64, Fig. 2b). Furthermore, when the ABZ- and MBZ-induced gene expression profiles were compared using the CMAP database, ABZ was only ranked number 222 using MBZ as the query signature (Fig. 2c). These results indicate differences in mechanism of action between ABZ and MBZ.

**Fig. 3:**
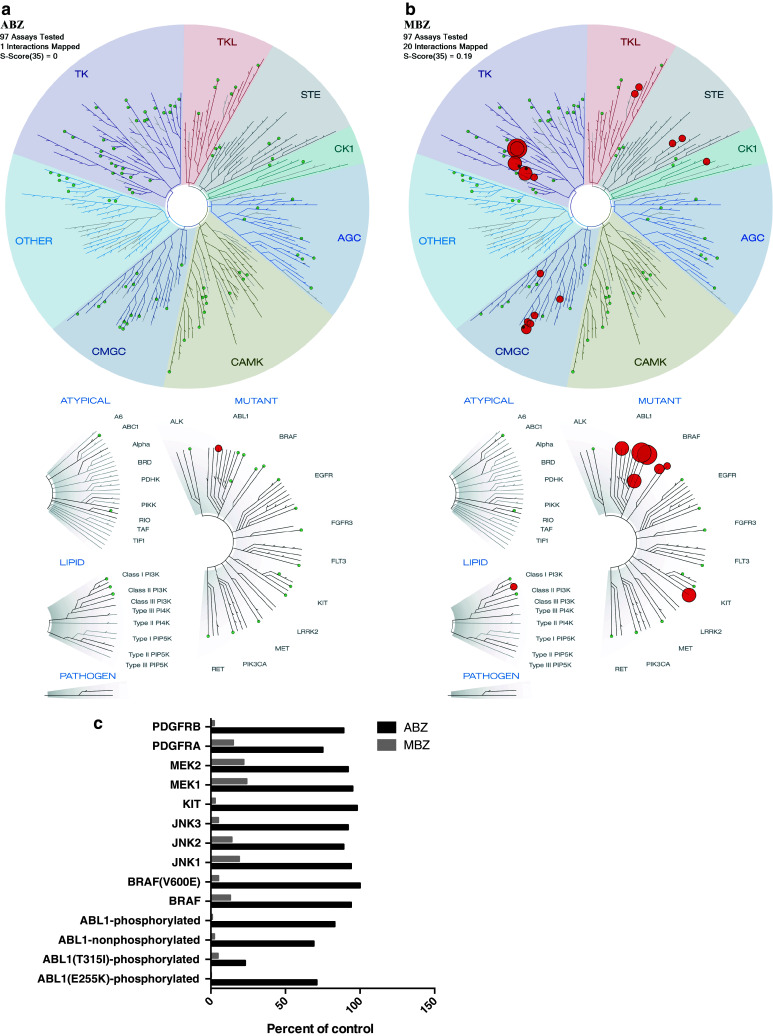
The binding affinities of ABZ and MBZ were tested at 10 μM in a high-throughput binding assay (KINOMEscan, Discoverx) against a panel of 97 kinases. In **a**, **b,** the treespot dendrogram for ABZ and MBZ are shown, respectively. The percent of control values <25 % for MBZ for selected cancer related kinases are shown in **c**

We subsequently tested the binding affinities of MBZ and ABZ at 10 µM against a panel of 97 kinases. Whereas no kinase binding was observed for ABZ, MBZ appeared to be a potent inhibitor of several kinases with high affinity (POC <22, Fig. 3). Binding constants (Kds) in response to MBZ were also determined for selected kinases (Table 1) Interestingly, MBZ showed strong activity with Kds in the nanomolar range against ABL kinases including the T315I gatekeeper binding site point mutation which remain a major clinical challenge in treatment for CML. Cells that harbor this mutation are insensitive to most of the clinically available ABL-targeted drugs. Furthermore, MBZ was also shown to potently bind to BRAF and especially to mutant BRAF(V600E), which is of clinical relevance in melanoma and colon cancer (Fig. 3; Table 1). In CMAP, the MBZ-induced gene expression profile correlated strongly with nocodazole a well-known tubulin inhibitor with chemical structure similarity to MBZ. Also nocodazole has recently been shown to inhibit several protein kinases including Bcr–Abl (Park et al. 2012). These results thus suggest additional potential targets of importance for MBZ efficacy.

**Table 1:** Binding affinities for mebendazole for various kinases

Kinases	POC (10 μM)^a^	Kd (μM)^b^
ABL1(E255K) phosphorylated	0.3	0.078
ABL1(T315I) phosphorylated	4.8	0.170
ABL1 nonphosphorylated	2.4	0.550
ABL1 phosphorylated	0.95	0.130
BRAF	13	0.190
BRAF(V600E)	5.1	0.250
JNK3	5.2	0.420
KIT	2.9	0.460
PDGFRA	15	0.340
PDGFRB	2.3	0.560

^a^Selected kinases with percent control (POC) values at 10 μM are shown

^b^Values are the average of two determinations

**Fig. 4:**
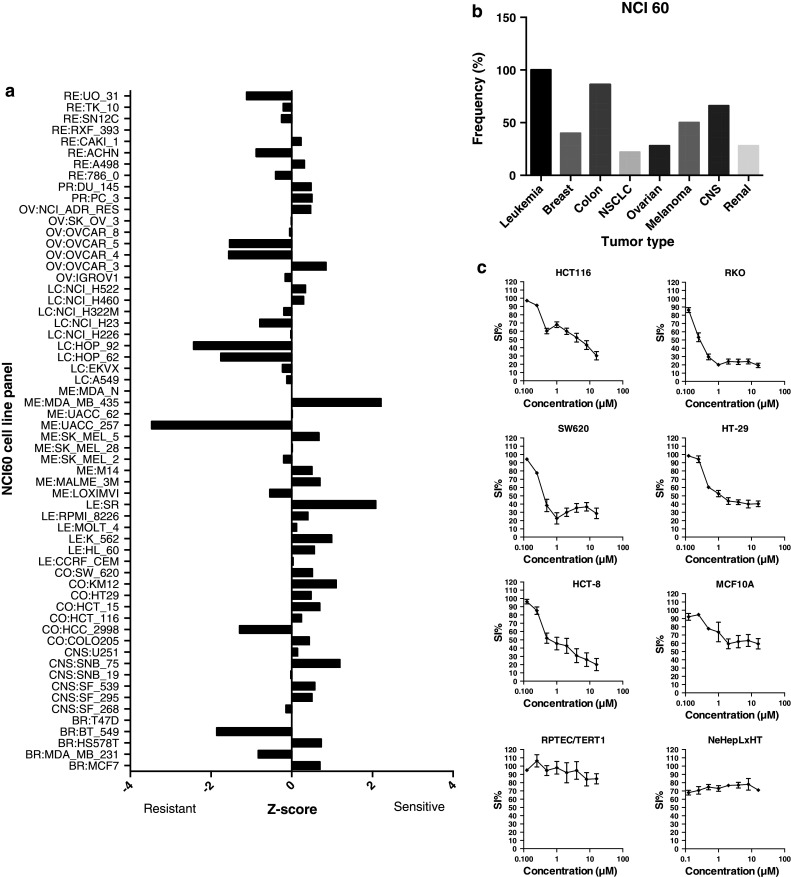
Analysis of NCI 60 data. The mean graph based on *z* scores for MBZ is shown in **a**. In (b), the frequency of positive *z* scores (sensitive cell lines) for all subpanels of the NCI 60 with more than 4 members is displayed. In **c,** concentration-dependent effects of MBZ on cell survival in five colon cancer cell lines (HCT 116, RKO, HT29, HT-8 and SW626) and in three cell lines with non-malignant phenotypes (epithelial MCF 10A, renal RPTEC/TERT1 and hepatocyte NeHepLxHT) are shown. Survival was determined over 72 h using the FMCA assay. The results are expressed as percentage of the untreated control and presented as mean values ± standard error of the mean from three independent experiments

Analysis of the diagnosis-specific activity of MBZ using NCI 60 *z* score data revealed 80 % of the colon cancer cell lines to be sensitive, second only to the leukemia panel (Fig. 4a). The lung and renal cancer subpanels showed the lowest frequency of sensitive cell lines, 25 and 28 %, respectively (Fig. 4b). The average GI 50 over the panel was 0.46 μM.

Five colon cancer cell lines, including those used in the primary screen, and three cell lines with non-malignant phenotypes were subsequently tested for response to MBZ (Fig. 4c). All 5 colon cancer cell lines showed a MBZ IC_50_ of <5 µM whereas the drug was largely inactive in the non-malignant cell line models, thus indicating a potentially good therapeutic index in colon cancer (Fig. 4c). Indeed as a first indication of this possibility, a patient with refractory metastatic colon cancer was treated with MBZ at the standard anthelmintic dose of 100 mg twice daily. The patient experienced no subjective adverse effects at all from the drug and computerized tomography evaluation after six weeks of therapy showed near complete remission of the metastases in the lungs and lymph nodes and a good partial remission in the liver (case report accepted for publication in Acta Oncologica).

In conclusion, the overall results indicate that MBZ have repositioning potential for treatment of advanced colon cancer and clinical trials seem warranted.
